# Chromosome level assemblies of *Nakaseomyces (Candida) bracarensis* uncover two distinct clades and define its adhesin repertoire

**DOI:** 10.1186/s12864-024-10979-8

**Published:** 2024-11-07

**Authors:** Marina Marcet-Houben, Ewa Księżopolska, Toni Gabaldón

**Affiliations:** 1 Barcelona Supercomputing Centre (BSC-CNS), Plaça Eusebi Güell, 1–3, Barcelona, 08034 Spain; 2 Institute for Research in Biomedicine (IRB Barcelona), The Barcelona Institute of Science and Technology, Baldiri Reixac, 10, Barcelona, 08028 Spain; 3 Centro de Investigación Biomédica En Red de Enfermedades Infecciosas (CIBERINFEC), Barcelona, Spain; 4 Catalan Institution for Research and Advanced Studies (ICREA), Barcelona, Spain

**Keywords:** Genomics, *Nakaseomyces*, Adhesins, Nanopore

## Abstract

**Background:**

The *Nakaseomyces* clade is formed by at least nine described species among which three can be pathogenic to humans, namely *Nakaseomyces glabratus* (*Candida glabrata*), the second most-common cause of candidiasis worldwide, and two rarer emerging pathogens: *Nakaseomyces (Candida) nivarensis* and *Nakaseomyces (Candida) bracarensis.* Early comparative genomics analyses identified parallel expansions of subtelomeric adhesin genes in *N. glabratus* and *N. nivarensis/bracarensis*, and suggested possible links with the emergence of the virulence potential in these species. However, as shown for *N. glabratus*, the proper assessment of subtelomeric genes is hindered by the use of incomplete assemblies and reliance on a single isolate.

**Results:**

Here we sequenced seven *N. bracarensis* isolates and reconstructed chromosome level assemblies of two divergent strains. We show that *N. bracarensis* isolates belong to two diverging clades that have slightly different genomic structures. We identified the set of encoded adhesins in the two complete assemblies, and uncovered the presence of a novel adhesin motif, found mainly in *N. bracarensis*. Our analysis revealed a larger adhesin content in *N. bracarensis* than previously reported, and similar in size to that of *N. glabratus*. We confirm the independent adhesin expansion in these two species, which could relate to their different levels of virulence.

**Conclusion:**

*N. bracarensis* clinical isolates belong to at least two differentiated clades. We describe a novel repeat motif found in *N. bracarensis* adhesins, which helps in their identification. Adhesins underwent independent expansions in *N. glabratus* and *N. bracarensis*, leading to repertoires that are qualitatively different but quantitatively similar. Given that adhesins are considered virulence factors, some of the observed differences could contribute to variations in virulence capabilities between *N. glabratus* and *N. bracarensis.*

**Supplementary Information:**

The online version contains supplementary material available at 10.1186/s12864-024-10979-8.

## Background

*Nakaseomyces* is a clade of Saccharomycotina yeasts, sister to the *Saccharomyces genus*, and currently formed by nine described species, of which six are environmental (*Nakaseomyces delphensis*, *Nakaseomyces castellii*, *Nakaseomyces bacillisporus*, *Nakaseomyces uthaithaninus*, *Nakaseomyces kungkrabaensis* and *Nakaseomyces sp. UFMG-CM-Y6046*) and three are opportunistic pathogens (*Nakaseomyces glabratus*, *Nakaseomyces nivariensis* and *Nakaseomyces bracarensis*) [1–5]. This clade has long been known to contain the important human pathogen *N. glabratus* (previously *Candida glabrata*). Molecular data shows that *N. glabratus* is more closely related to *Saccharomyces cerevisiae* than the human pathogenic species *Candida albicans* and therefore Kurtzman renamed the species to *Nakaseomyces *[6]. An effort is being made to adopt this nomenclature, which we will use in this manuscript. *N. glabratus* is one of the major causal agents of candidiasis, following *Candida albicans*, whereas *N. nivariensis* and *N. bracarensis* are considered emerging pathogens and have often been misidentified as *N. glabratus* [7–10]. Given the difficulties of distinguishing between *N. glabratus* and the other two species, the assessment of their incidence is inaccurate. Nevertheless, several studies have re-analysed large samples of *N. glabratus* isolates to identify the presence of *N. bracarensis* and *N. nivariensis*. In a global analysis of 1,598 strains identified as *N. glabratus* by routine methods, 0.2% of the strains were found to be either *N. bracarensis* or *N. nivariensis* [11]. This trend is also found in other, more specific studies, where few samples of non-glabratus *Nakaseomyces* pathogens are found [10]. For instance, in a set of 353 *N. glabratus* isolates from Poland, only one non-glabratus isolate was found that corresponded to *N. bracarensis* [12]. In a different analysis of 137 strains of *N. glabratus*, three *N. bracarensis* isolates were identified [13]. And from a set of 8,000 samples of *N. glabratus*, 12 *N. nivariensis* and 1 *N. bracarensis* strains were identified in China [14]. Despite their low incidence, Kumar et al. [15] showed that the presence of these two pathogens, as assessed from the literature, has increased during the last ten years, a trend common to other rare pathogenic species in the Saccharomycotina.

Little is known about the virulence mechanisms governing pathogenesis in *N. bracarensis*. It is possible that many of the virulence factors that have been studied in *N. glabratus* also play an important role in the virulence of *N. bracarensis*. Such methods include having a large content of adhesin genes, being able to secret proteases and phospholipases, being able to build biofilms or other, more complex mechanisms, that are geared to increase drug resistance such as the overexpression of drug transporters, mutations targeting genes involved in the action mechanisms of said drugs and others [16–19]. Yet, infection experiments using the model worm *Caenorhabditis elegans* showed that *N. bracarensis* is the least virulent of the three pathogenic *Nakaseomyces* species [20], which is congruent with its comparatively lower incidence [10] and also points to the likelihood of virulence mechanisms being different in the three *Nakaseomyces* species. Until now little has been done to pinpoint such differences using experimental approaches, Moreira et al. assayed three *N. bracarensis* isolates and determined that they all could form biofilms to various degrees [21]. They also showed that only one of the strains had proteolytic activity and that they were unable to produce phospholipases. Lima et al. showed that the cell wall of *N. bracarensis* had a higher porosity which has been linked to a larger accessibility to β-1,3-glucans and a less successful masking of the pathogen from host immune recognition [18, 22].

More attention has been paid to the genomic differences among the *Nakaseomyces* species. The genome of *N. bracarensis* was first sequenced 10 years ago in a comparative genomics analysis of the six *Nakaseomyces* species that were known at that time [3]. In that analysis, the genomic basis for virulence was explored. One relevant result was the finding that *N. bracarensis* had 12 *EPA*-like adhesins. *EPA* stands for Epithelial adhesins and these genes are considered to be important virulence factors in *N. glabratus*. The amount of *EPA* adhesins in *N. bracarensis* was less than the 30 found in *N. glabratus* but more than the single *EPA* gene found in the non-pathogenic relative *N. delphensis*. A detailed phylogenetic analysis of the *EPA* family of adhesins showed independent gene family expansions in *N. glabratus* as compared to the other two pathogens. This led to the hypothesis that the independent expansions of *EPA*-like adhesin content in *N. glabratus* and the other two pathogenic species played an important role in the emergence of virulence in the *Nakaseomyces*. More recently, a re-sequencing analysis of *N. nivariensis* including long-read data obtained a more accurate account of the adhesin complement in this species, increasing the predicted number from 10 to 53 [23]. Such discrepancy was attributed to differences in the prediction method coupled with the fact that the initial assembly had been based only on short read data [23]. In addition, in a recent analysis of 21 chromosome-level assemblies in *N. glabratus*, we showed that at least 10% of adhesins went undetected when using only short read based assemblies [24]. Also, different strains of *N. glabratus* had slightly different sets of adhesins. Experimental validation of adhesins has so far been sparse, with only a few specific families being studied in depth in *N. glabratus* (EPA family, AWP family and PWP family) [25–29], therefore knowledge on this important virulence factor relies so far mostly on bioinformatic predictions. This underscores the importance of using long-read based assemblies when predicting adhesin genes in *Nakaseomyces* species, as well as the need to assess diverse strains to obtain the full breadth of putative adhesin content in a given species. This has prompted us to re-sequence the genome of *N. bracarensis* using long read sequencing technology and sequence a set of 7 strains to accurately establish the adhesin catalogue in this poorly known emerging pathogen.

## Results and discussion

### Assembly and comparison of *Nakaseomyces bracarensis* genomes

**Fig. 1:**
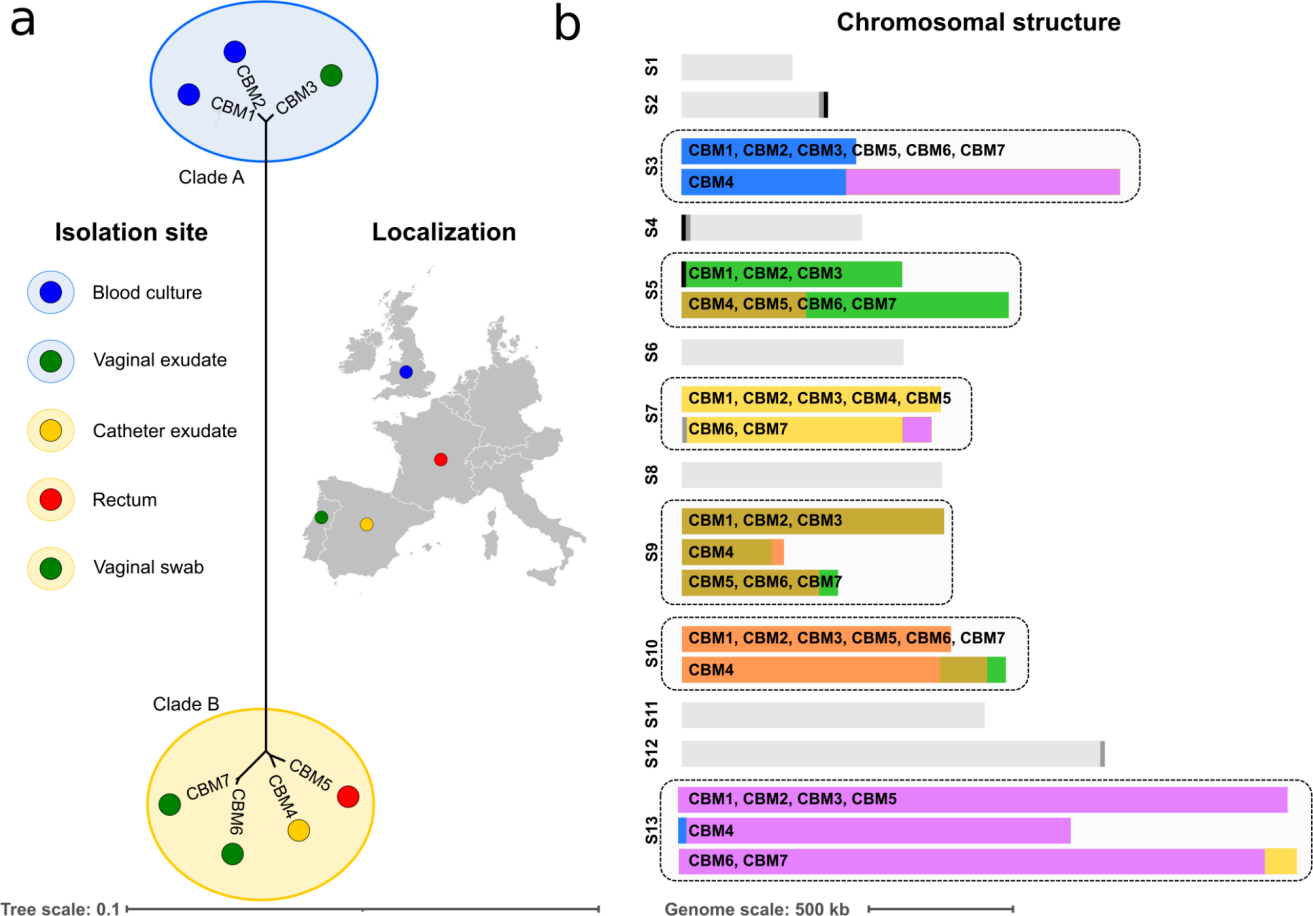
(**a**) Strain tree showing the seven *N. bracarensis* isolates sequenced in this study. Each strain is represented in the tree by a colored circle, and each clade is indicated by a light colored background. The combination of circle color and background indicate the body site (left) and the country (map on the right) of isolation. The bar located at the bottom of the tree indicates the tree scale in terms of inferred substitutions per site. (**b**) Graph representing the translocations found among strains of *N. bracarensis*. The 13 scaffolds are represented in two different ways. If the scaffold is represented as a single grey bar, this scaffold has not undergone any translocation. If the scaffold is represented by multiple, colored bars, then each of the bars represents one of the structural variants found in the strains. On top of each colored bar is the list of strains that have that variant in its genome. Bar lengths are always proportional to the scaffold size. Missing telomeres are marked with a black (CMB3) or dark grey (CBM6) bar at the end of the Scaffolds. The bar at the bottom of the image indicates the scale in terms of 500 Kb of the chromosome bars

Seven strains of the haploid species *N. bracarensis* were sequenced using illumina technology. Two pairs of strains were of clonal origin but presented different morphologies (CBM1 / CBM2 and CBM6 / CBM7) (Table 1). Reads of each of the seven strains were mapped to the previously available reference genome [3]. A pseudo alignment was then built taking the reference genome and substituting positions with Single Nucleotide Polymorphisms (SNPs). Then a phylogenetic tree was built based on this alignment (see methods). The resulting tree showed that the strains were clearly divided into two different clades which we named A and B (see Fig. 1A). We choose one representative isolate for each clade for long-read sequencing: CBM3 (reference genome of the type strain CBS10154) for clade A, and CBM6 for clade B. The genomes of these two isolates were assembled combining short reads and nanopore long reads using the longHam pipeline (see Materials and Methods). The resulting assembly statistics (Table 2) indicate a high contiguity and completeness, and show a clear improvement in CBM3 when compared to the previously available assembly. *N. bracarensis* was previously predicted to have 12 chromosomes which would imply a chromosome split in both assemblies [3]. 86% of the telomeric regions for the two genomes of *N. bracarensis* were found. We tried to join contigs that did not contain a telomeric region but were unable to find nanopore reads that could reliably join two contigs. The lack of telomeres in some of the scaffolds was strongly associated with the presence of the ribosomal repeats containing 5.8 S rRNA, 18 S rRNA and 25 S rRNA. Those clusters were located at one end of Scaffolds 2, 4 and 5 in CBM3, all of which lacked telomeres. In CBM6 they were located in Scaffolds 2, 4 and 9, of which only Scaffold 9 had the two telomeres. Considering that both *N. glabratus* and *N. nivariensis* have 13 chromosomes, one possibility is that *N. bracarensis* could also have 13 chromosomes and that we are missing the telomeric repeats region following the presence of ribosomal repeats. Alternatively, the ribosomal repeats region could extend to such a long region that nanopore reads are not able to cover the whole cluster and a chromosome is therefore split in two.

**Table 1:** List of strains analysed in this study

Strain code	Strain name	Origin (tissue)	Country	Publication	Comments
CBM1	NCYC 3133	Blood culture	UK	[5]	Small colony
CBM2	NCYC 3133	Blood culture	UK	[5]	Large colony
CBM3	CBS10154	Vaginal exudate	Portugal	[5]	Type strain
CBM4	CL7030	Catheter exudate	Spain	[30]	
CBM5	445 L	Rectum	France	[22]	
CBM6	246,188	Vaginal swab	Portugal	[22]	Small colony
CBM7	246,188	Vaginal swab	Portugal	[22]	Large colony

**Table 2:** Genome assembly statistics of the two sequenced strains of *N. bracarensis* and the genome of the type strain assembled in 2013

	*N*. bracarensis reference 2013 (type strain)	*N*. bracarensis CBM3 (type strain)	*N*. bracarensis CBM6
NCBI BioProject code (genome accession code)	PRJEB145 (CAPU00000000.1)	PRJNA1001830 (JBEVYE000000000)	PRJNA1001830 (JBEVYD000000000)
Genome size (Mbp)	12,41	12.74	12.75
Number of contigs	40	13	13
Number of contigs (> 100.000 bp)	20	13	13
N50 (bp)	757,353	936,813	1,085,018
GC content	0.361	0.368	0.369
Number of Ns	182,685	1850	0
Number of Telomeres	3	23	22

We then mapped the short reads from the remaining five strains to the newly assembled reference genome (strain CBM3) in order to assess genetic variants, including structural differences. We validated these variants by mapping these reads to the CBM6 assembly. As previously observed, strains could be divided into two different clades according to the number of SNPs with respect to the reference. CBM1 and CBM2 were close to the CBM3 reference with 0.07 SNP / Kb whereas the other strains were farther apart with 1.7 SNP / Kb. More variation was found when considering large structural variants (see Fig. 1B). As expected CBM1 and CBM2 had nearly the same structure as CBM3 and only a few duplications and deletions were found based on read coverage depth (32 deleted regions and 20 duplicated regions). The strains belonging to Clade B contained higher numbers of deleted and duplicated regions (ranging from 36 to 56 deleted regions and from 32 to 99 duplicated regions). All members of Clade B also shared a translocation between Scaffolds 5 and 9. In addition CBM6 and CBM7 shared a translocation between scaffolds 7 and 13, and CBM4 had translocations between scaffolds 3 and 13 and between scaffolds 9 and 10. Translocations have been shown to be common among strains of *N. glabratus* and it seems this closely related species also contains this tendency towards plasticity which has previously been related to antifungal drug resistance [24, 30, 31].

Based on read mapping we assessed whether any of the genes encoded in CBM3 were missing in any of the other strains. In total we found 20 genes that were absent or partially truncated in at least one of the other strains (see Supplementary Table 1). Among those we found nine genes coding for adhesins, two putative transcription factors and one Beta-mannosyltransferase (BMT). In the genome of *N. glabratus* there are seven BMTs, five of them located in a gene cluster. Interestingly Jawhara et al. [32] showed that the deletion of this cluster affected the mannoprotein content in the fungal cell wall and decreased virulence in a murine model treated with dextran sulfate sodium (DSS) to induce *N. glabratus* infection. Compared to *N. glabratus*, *N. bracarensis* CBM3 has six BMTs and only two of those are contiguous in the genome. One of the BMTs present in CBM3 is missing in all Clade B strains (CBM3_13_05163). When the analysis was done using CBM6 as a reference less proteins appeared lost in other strains. Two adhesins lost in CBM5 had already been predicted in the previous analysis. The remaining four losses were not found in any of the strains of clade A and two of them were adhesins.

### Adhesin detection and discovery of a novel repetitive motif

The adhesin complement of a strain is better determined on genomes assembled using long-read data [24]. Hence, we selected the two strains of *N. bracarensis* for which we have long-read based assemblies (CBM3 and CBM6) which also represent each of the two identified clades. For both genomes we applied a pipeline previously designed to detect putative adhesins in *N. glabratus* [24]. In addition, glycosylphosphatidylinositol (GPI)-anchors were searched for using NetGPI [33].

*N. glabratus* putative adhesins are known for having repetitive regions, which often contain several VSHITT and SFFIT motifs. When those motifs were described they were found to be unique for *N. glabratus*. Given the predicted set of adhesin genes detected in *N. bracarensis* we searched for those two motifs. Only the SFFIT motif was found repeated in one protein in each of the *N. bracarensis* strains which is far from the 16 proteins with repeated SFFIT motifs and 36 proteins with repeated VSHITT motifs found in the *N. glabratus* reference strain. This supports the hypothesis that adhesins evolved independently in *N. bracarensis* and *N. glabratus* [3].

We then searched whether *N. bracarensis* also had adhesin specific repetitive motifs that could be used to help identify additional putative adhesins. We used the previously predicted adhesins to identify all motifs of 5, 6 and 7 amino acids that were found in at least 10 of the adhesins in one strain. We obtained a single motif, SDGKTHT, that was found in 31 of the adhesins in both strains of *N. bracarensis* and in all of them it was repeated at least twice and up to 228 times (in protein CBM6_11_03410). When searched in other *Nakaseomyces* genomes, we found that the motif was also repetitive in a few other proteins (5 with up to 12 repeats) of the emerging pathogen *N. nivariensis*. In contrast the motif was absent in *N. glabratus*, *N. delphensis*, *N. bacillisporus* and *N. castellii*. We extended the search for the presence of the motif to the OMA database, as it contains an extensive set of species (more than 14 million proteins for 2,282 species) [34]. The motif was found in only six proteins but in none of those the motif was repeated. This means that this motif is exclusive for adhesins in some *Nakaseomyces* species, mainly *N. bracarensis*, just as VSHITT and SFFIT had been previously described in *N. glabratus*. Proteins that contained the SDGKTHT motif and had not been included in the list of predicted adhesins were also added to the list. The full list of predicted adhesins for strains CBM3 and CBM6 can be found in Supplementary Tables 2 and 3 respectively.

We then further explored the properties of the identified motifs. We found no clear tendency with respect to their relative position within the protein sequences, and this was also the case when reproducing this analysis in *N. glabratus* with the VSHITT and SFFIT motifs. It is hypothesized that the large stretches of VSHITT and SFFIT motifs found in the adhesins of *N. glabratus* and the resulting high density of S/T residues result in a more glycosylated cell wall as compared to the one of *S. cerevisiae* [18]. We used the GlycoEP web-server to predict N-linked and O-linked glycosylation sites in *N. bracarensis* putative adhesins containing the SDGKTHT motif [35]. On average 18% of the amino acids were predicted to contain O-linked glycosylated sites. Other predicted adhesins and five random sets of 30 proteins gave much lower glycosylation levels (10% and an average of 2% respectively). This difference was not observed when predicting N-linked glycosylation sites (putative adhesins with the motif: 4%, putative adhesins without the motif: 5%, random set: 5%). Similar results were found for putative adhesins of *N. glabratus* containing VSHITT or SFFIT motifs when compared to putative adhesins without these motifs (14% versus 9%). This suggests that the presence of the SDGKTHT motif contributes to the O-linked glycosylation patterns as reported for the adhesin motifs in *N. glabratus*. The presence of repetitive motives in predicted adhesin genes has been correlated with increasing adhesion and flocculation abilities, which will need to be further explored in the case of *N. bracarensis* [36]. In addition, differences in glycosylation patterns due to the presence of a different repetitive motif could be linked to the differences in virulence observed between *N. glabratus* and *N. bracarensis*. All these inferences, including the predictions of adhesins have yet to be experimentally validated but this analysis opens the door to understand key differences in virulence patterns between *N. glabratus* and *N. bracarensis*.

### Evolution of adhesins in pathogenic *Nakaseomyces* species

To see how the different predicted adhesins have evolved in *N. bracarensis* when compared to *N. glabratus* we joined the predictions found in the two strains of *N. bracarensis* and added a representation of adhesins of *N. glabratus*. This last set is formed by one representative member of the previously determined adhesin families in this species [24]. Adhesins from the reference (CBS138) [37] or from strain BG2 [38], the two strains in *N. glabratus* with the best studied adhesin complement, were chosen as the representative whenever possible (see Supplementary Table 4). Interestingly, *N. bracarensis* CBM3 and CBM6 encoded 72 and 66 putative adhesins respectively, which is comparable to the number of adhesins found in strains of *N. glabratus*, which ranged between 67 and 81 [24]. This is in contrast with predictions made based on short read genomes which indicated that *N. bracarensis* had fewer adhesins than *N. glabratus* [3]. For each predicted adhesin we limited the protein sequence to the first 300 residues and then clustered the adhesins using MCL [39]. This divided the putative adhesins into twelve different families for which we built phylogenetic trees for those that had at least four sequences (families 1 to 9) (see Table 3). While in most adhesin families all species were represented, the phylogenetic reconstructions indicated that each family had expanded independently in the different species.

**Table 3:** List of adhesins families inferred for *N. bracarensis* and related to adhesins known in *N. Glabratus*

Family number	Family name	*N*. glabratus homologs
1	*EPA*	*EPA1* to *EPA 26*
2	*AWP12* homolog	*AWP12*, *CAGL0C01133g*, *CAGL0C00825g*, *CAGL0C00803g*, *CAGL0C00858g*, *CAGL0C00968g*, *NAO1*
3	*AWP2 / 4* homolog	*AWP2a* to *AWP2i*, *AWP4*, *CAGL0I07293g*, *CAGL0E06600g*
4	*AED1 / AED2* homolog	Specific for *N. glabratus* *AED1*, *AED2*, *AWP13*, *NAO2*, *NAO3*, *NAO4*, *QNG13707.1*, *CAGL0L00157g*, *CAGL0J00132g*, *CAGL0G00099g*, *CAGL0A04851g*, *CAGL0E00231g*, *CAGL0E00165g*, *CAGL0L10092g*, *CAGL0E01661g*, *CAGL0C00253g*, *CAGL0C03575g*
5	*PWP*	*PWP1* to *PWP7*
6	*AWP1* homolog	*AWP1*, CAGL0J01727g, CAGL0J01800g, CAGL0J02530g, CAGL0J02552g, CAGL0J01774g
7	Family 7	*CAGL0J05159g*, *CAGL0L09911g*, *CAGL0G04125g*
8	*AWP3* homolog	*AWP3a* and *AWP3b*
9	Family 9	Specific to *N. bracarensis*
10	Family 10	Specific to *N. bracarensis*
11	Family 11	Specific to *N. bracarensis*
12	*AWP6 / AWP7* homolog	Specific for *N. glabratus* *AWP7*, *AWP6*, *CAGL0E00187g*

**Fig. 2:**
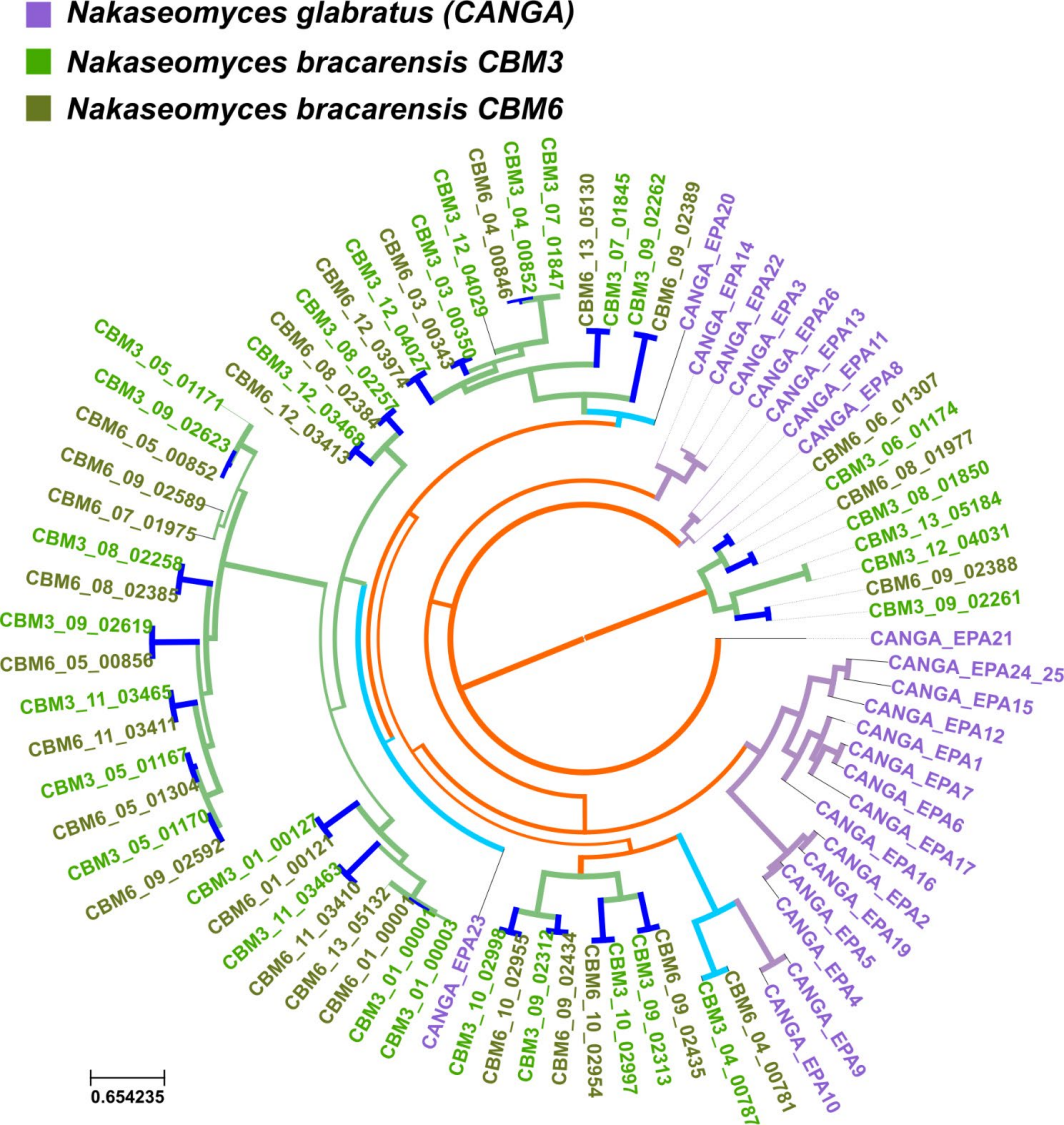
Phylogenetic tree representing the evolution of the EPA gene family in *N. glabratus* and *N. bracarensis*. Names in purple indicate genes of *N. glabratus* strains (identified with the tag CANGA) and names in shades of green indicate genes of *N. bracarensis* (identified as CBM3 or CBM6 depending on the strain). Branches are colored in purple when a duplication event has been detected that is specific for *N. glabratus*, in green when the duplication is specific for *N. bracarensis*, in orange when the duplication is ancestral to the divergence between both species and finally in blue when there is a speciation event (i.e. no duplication). Light blue indicates speciation events between *N. glabratus* and *N. bracarensis* whereas dark blue indicates divergence between the two strains of *N. bracarensis*. Nodes with less than 50% bootstrap support are collapsed. The thickness of the branch correlated with the node support

The largest family contains the *EPA* gene family, some of which are known to mediate adhesion to different types of cells, among which are epithelial cells (*EPA1*, *EPA6* and *EPA7* primarily) and are considered a virulence factor in *N. glabratus* [19]. In a previous report based on short read genome sequencing it was shown that this family was expanded independently in the different species and additionally was more often found in *N. glabratus* (25 *EPA*s) [3]. Our analysis, based on long read assemblies, shows that *N. bracarensis* is in fact the species with the largest amount of *EPA* genes with 30 and 27 putative adhesins found in strains CBM3 and CBM6 respectively. Interestingly, few *EPA* genes in *N. glabratus* had orthologs in *N. bracarensis* (see Fig. 2). *N. glabratus EPA9* and *EPA10*, which are closely related paralogs, have an ortholog in *N. bracarensis*. And, interestingly, orthologs to *N. glabratus EPA20* and *EPA23* have been extensively expanded in *N. bracarensis* species. According to glycan binding profiles performed in *N. glabratus* by Diderrich et al., each *EPA* shows a specific binding pattern pointing to functional differences among the different genes [40]. When clustering *EPAs* based on their different binding affinities, *EPAs* were classified in three different groups. *EPA9* and *EPA10* belong to class I, and share this class with *EPA1*. When tested to determine cell adhesion caused by each specific *EPA* gene by introducing a plasmid containing a specific *EPA* gene to a *Saccharomyces cerevisiae* model, *EPA9* was classified as causing medium adherence when compared to *EPA1*, whereas *EPA10* caused a lower adherence [40]. The fact that *N. bracarensis* strains have orthologs to *EPA9* and *EPA10* instead of the more adherent *EPA1* gene could also be related to the lower incidence of this emerging pathogen in the clinic. Still, experimental evidence of the role of *EPA9* and *EPA10* will need to be provided to see the impact those genes have on adhesion in *N. bracarensis*. On the other hand, *EPA23* belongs to class II, that best binds to sulfated galactosides. Like *EPA9*, it had medium adherence in the in-vivo adherence tests performed. Finally, *EPA20* belongs to class III, a group of *EPAs* with less specific bindings but that have a tendency to bind to acidic sugars, in addition it has a low binding affinity to human epithelial cells. Little is known about the role of *EPA20* or *EPA23* in *N. glabratus*. In two different studies which created hypervirulent strains of *N. glabratus* through the mutation of different genes, both *EPA* genes were upregulated [41, 42] which could indicate a direct role in virulence for these *EPAs* but further experimental studies on why this particular genes have been expanded in *N. bracarensis* need to be performed.

**Fig. 3:**
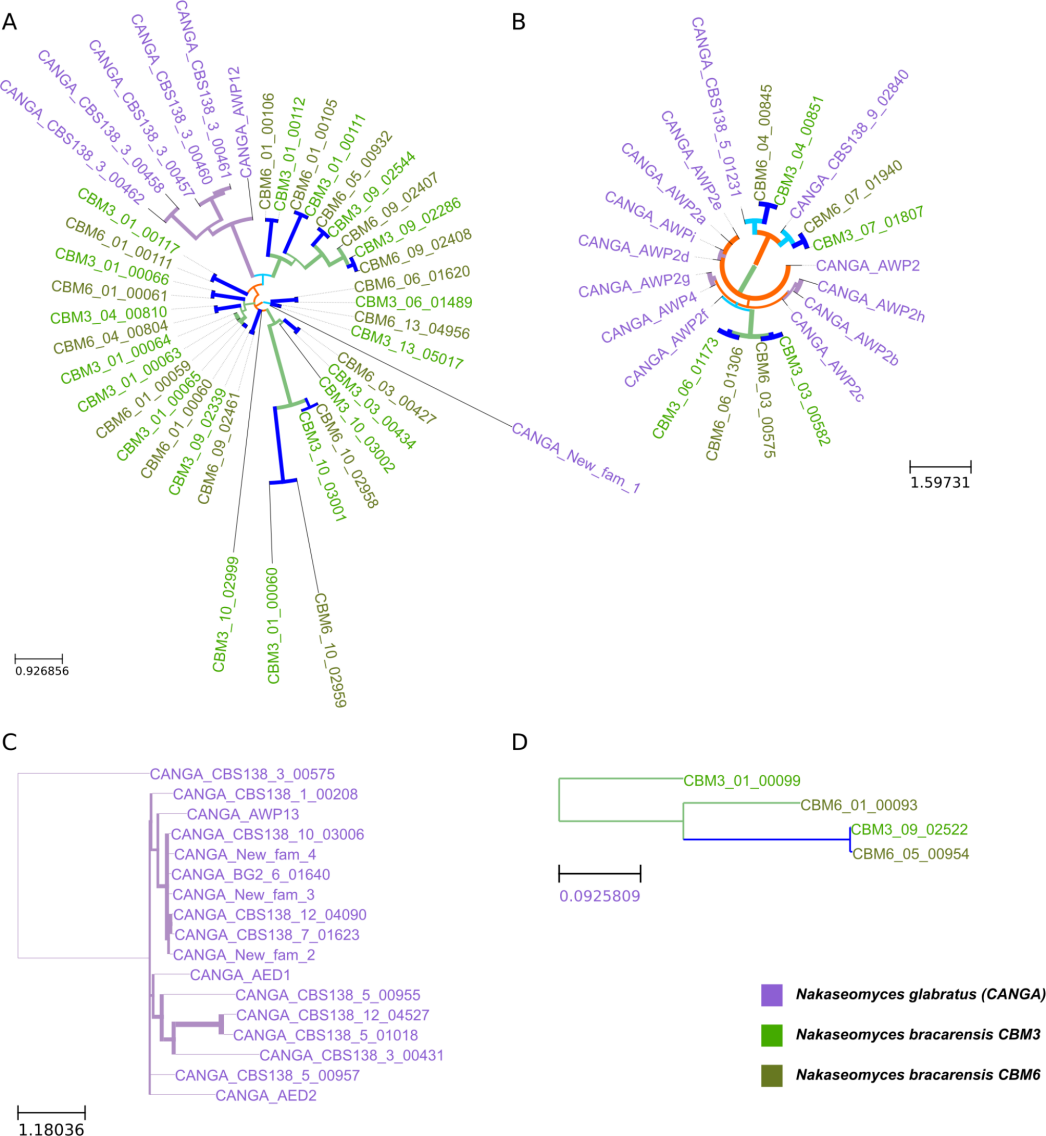
Phylogenetic trees of different families of adhesins. (**A**) Adesin family containing AWP12 and expanded in N. bracarensis. (**B**) AWP2 family. (**C**) Adhesin family containing AED1 and AED2, specific for *N. glabratus*. (**D**) Adhesin family 9, specific for *N. bracarensis*. Trees are colored as in Fig. 2

Other families of adhesins show different evolutionary histories (see Fig. 3 and Supplementary Fig. 1). For instance, the second largest cluster of adhesins derived from this analysis contains *N. glabratus* gene *AWP12* which has been found related to biofilm formation (see Fig. 3A) [19]. Orthologs in *N. bracarensis* have expanded independently, having 18 copies in *N. bracarensis* CBM3 and 15 in *N. bracarensis* CBM6. The third largest cluster contains 13 genes belonging to *N. glabratus*, among which are the 11 members that form family *AWP2*. *N. bracarensis* has only four genes in this family, of which two are orthologs to *AWP2f* (see Fig. 3B). Five of the adhesin families are species-specific. For instance, the family containing *AED1* and *AED2* (see Fig. 3C) and the one containing *AWP6* and *AWP7* were specific for *N. glabratus* whereas families 9 (see Fig. 3D), 10 and 11 were specific for *N. bracarensis*. This analysis shows that, while the number of adhesins between *N. glabratus* and *N. bracarensis* is comparable, their evolution has followed independent paths, which could be a factor accounting for their differences in virulence and clinical prevalence.

## Conclusions

We here present the first long-read assemblies for *N. bracarensis* and a first population genomics analysis of this poorly known emerging pathogen. Our results show that there are at least two clades in this species with a sequence divergence of roughly 0.17% at the nucleotide level and having some large genomic re-arrangements and gene content differences. Like in *N. glabratus*, part of the gene content differences are related to adhesins.

Two of the strains of *N. bracarensis* were assembled using long read technology which provided a near chromosome level assembly. Using these genomes we performed a comparative analysis of adhesin presence in *N. bracarensis* and *N. glabratus*. Notably, when analysing the sequence of adhesins in *N. bracarensis*, we observed a recurring motif (SDGKTHT) in many of them, similar to the motifs VSHITT and SFFIT described in *N. glabratus*. The SDGKTHT motif was therefore used to aid in the search of adhesins in this species. Unlike what had been previously reported, based on a more fragmented genome, *N. bracarensis* has a similar amount of adhesins as compared to *N. glabratus*, underscoring the importance in assembling genomes using long reads to detect these elusive subtelomeric genes. Phylogenetic analysis of adhesin families showed different evolutionary patterns. Specifically, *EPA* genes were duplicated independently in the two species, being *EPA20* the most expanded gene in *N. bracarensis*. Orthologs to *EPA9* and *EPA10* which have been associated with adherence to epithelial cells though with a lower binding affinity than *EPA1*, might be linked to virulence in *N. bracarensis* though this and other observations found in the initial analysis of adhesins in *N. bracarensis* will need to be confirmed experimentally.

## Methods

### Strains and sequencing

We sequenced seven strains of *N. bracarensis* identified as CBM1 to CBM7 using short read technology (Table 3) [5, 22, 43]. Strains were stored in 50% glycerol at -80 C. Culturing of the strains involved plating a smear of biomass on YPD agar plates and incubating for 2 days at 30 ºC. Single colonies were then placed on 15 ml liquid YPD and incubated shaking at 30 ºC overnight.

Genomic DNA was extracted with MasterPureYeast DNA extraction kit (Epicentre, Cat. No. MC85200) and cleaned with Genomic DNA Clean & Concentrator (ZymoResearch Cat. No. D4065).

Illumina whole-genome sequencing was performed at the Genomics Unit from the Center for Genomic Regulation. Libraries were prepared using the NEBNext^®^ DNA Library Prep Reagent Set for Illumina^®^ kit (New England BioLabs) according to the manufacturer’s protocol. Briefly, 1 µg of genomic DNA was fragmented by nebulization in Covaris to ∼600 bp and subjected to end repair, addition of ‘A’ bases to 3′ ends and ligation of Truseq adapters. All purification steps were performed using QIAquick PCR purification columns (Qiagen). Library size selection was done with 2% low-range agarose gels. Fragments with average insert size of 700 bp were cut from the gel, DNA was extracted using QIAquick Gel extraction kit (Qiagen) and eluted in 30 µl EB. Ten microlitres of adapter-ligated size-selected DNA were used for library amplification by PCR using the Truseq Illumina primers. Final libraries were analyzed using Agilent DNA 1000 chip to estimate the quantity and check size distribution and were then quantified by qPCR using the KAPA Library Quantification Kit (KapaBiosystems, ref. KK4835) prior to amplification with Illumina’s cBot. Libraries were loaded at a concentration of 2 pM onto the flow cell and were sequenced 2 × 125 bp on Illumina’s HiSeq 2500.

Additionally for strains CBM3 (reference strain) and CBM6 we obtained long-read data with nanopore technology. Genomic DNA was extracted from *N. bracarensis* isolates using MasterPure Yeast DNA extraction kit (Epicentre, cat: Cat. No. MC85200) combined two extra RNA treatment (with Rnase A + T) with phenol-chloroform purification. Briefly, an overnight culture was pelleted, lysed with the Yeast Cell Lysis solution and treated with RNase provided by the kit. The samples were treated with MPC Protein Precipitation reagent (also from the kit) and the supernatant was then treated additionally for 30 min at 37 C with the Rnase A + T. The purification of the samples was done by phenol-chloroform purification and the DNA precipitation was done with 0.1 V Sodium Acetate 3 M and 2.5 V of cold EtOH 100%. To eliminate remaining RNA from the extraction, a third RNAse A + T treatment was done (2 h at 37 C) followed by DNA precipitation.

Nanopore sequencing was performed at the Ultra-sequencing core facility of the Centro Nacional d’Analisi Genòmica (CNAG) in Barcelona (Spain). Integrity of genomic DNA was analyzed by pulse field electrophoresis (Pippin Pulse, Sage Science), and contamination of DNA samples was checked with NanoDrop (Thermo Fisher Scientific) spectrophotometry based on 260/280 and 260/230 ratios. Then, the samples were used to prepare ten 1D2 and 1D genomic libraries using the Ligation sequencing kits SQK-LSK308 and SQK-LSK108 for sequencing on a MinION instrument (Oxford Nanopore Technologies, ONT). Two µg of genomic DNA were nick-repaired using the NEBNext FFPE DNA Repair Mix (NEB, M6630) and purified with 0.4X Agencourt AMPure XP Beads (Beckman Coulter, A63882). Samples were end-repaired and dA-tailed using the NEBNext UltraII End Repair/dA-Tailing Module (NEB, E7546) and subsequently purified with 1X Agencourt AMPure XP Beads. When using the SQK-LSK108 kit, the 1D sequencing Adapter Mix (AMX1D, ONT) was ligated to the purified samples using the Blunt/TA Ligase Master Mix (NEB, M0367L). When using the SQK-LSK308 kit, two adapter ligation steps were required. First, the 1D2 adapter was ligated to the purified samples using the Blunt/TA Ligase Master Mix. After a 0.4X purification with Agencourt AMPure XP Beads, the product with the 1D2 adapter was ligated to the BAM sequencing adapter (ONT) using the Blunt/TA Ligase Master Mix. The adapter-ligated products from both kit versions were purified using 0.4-fold excess of AMPure XP beads. The beads were washed twice using the Adapter Bead Binding Buffer (ABB, ONT), and the libraries were eluted in 15 µl of Elution Buffer (ELB, ONT).

Libraries prepared with the SQK-LSK308 kit were loaded into R9.5 or R9.5.1 chemistry FLO-MIN107 flow cells (ONT), and libraries prepared with the SQK-LSK108 kit were loaded into R9.4 chemistry FLO-MIN106 flow cells (ONT) according to manufacturer’s recommendations. In brief, first, the MinKNOW interface QC (ONT) was run to assess the flow cell quality, and this was followed by the flow cell priming. The sequencing library was mixed with a running buffer, Library Loading Beads (ONT), and nuclease free water and loaded onto a “spot on” port for sequencing. Sequencing data was collected during 48 h. The quality parameters of the sequencing runs were further monitored by the MinKNOW platform while the run was base-called using the Albacore 2.3.1.

### Assembly and read mapping

Sequencing reads from the seven strains of *N. bracarensis* were mapped to the previously published reference [3] using PerSVade v1.02.4 [44] to call single nucleotide polymorphisms (SNPs). Pseudo-sequences were built for each strain by substituting bases in the reference assembly with the corresponding predicted SNPs. These pseudo-sequences were used to obtain a strain based phylogenetic tree by selecting positions that contained a SNP in at least one of the strains and that had more than a 20 read coverage in all strains. The resulting alignment was used to build a strain tree using IQTREE v1.6 [45] allowing the program to select the best model.

Strains CBM3 and CBM6 were selected for long read sequencing as representatives of their respectives clades. CBM3 was chosen because it is the *N. bracarensis* type strain and CBM6 was chosen because in a preliminary analysis based on short read data it presented the highest amount of adhesins (results not shown). Strains CBM3 and CBM6 were assembled using the longHam pipeline v 1.0 (https://github.com/Gabaldonlab/longHam) [24]. Nanopore reads were limited to a random set of reads covering a 90X coverage of reads of at least 5000 bp. Reads were then corrected using the Canu v1.8 [46] correction process. Corrected reads were then limited to a coverage of 30X. LongHam then constructs a set of primary assemblies and then tests different combinations using Ragout v2.0 [47] to produce a final assembly that then is polished by pilon v1.22 [48]. Program versions and parameters for primary assemblies were as follows: (A) MaSuRCa v3.4.2 [49] (parameters: default); (B) Canu v1.8 [46] (parameters: default); (C) WTDBG2 v2.1 [50] (parameters: default); (D) a combination of Illumina assembly with platanus v1.2.4 [51] (parameters: default) and DBG2OLC v20180222 [52] (parameters: k 17 AdaptiveTh 0.01 KmerCovTh 10 MinOverlap 100 RemoveChimera 2 ContigTh 2), and (E) a combination of SparseAssembler v20160205 [53] (parameters: LD 0 k 51 g 15 NodeCovTh 1 EdgeCovTh 0) and DBG2OLC v20180222 [52] (parameters: k 17 AdaptiveTh 0.01 KmerCovTh 10 MinOverlap 100 RemoveChimera 2 ContigTh 2). For strain CBM3 the final assembly was a combination of the primary assemblies built with WTDBG2, DBG2OLC plus platanus and Canu whereas the CBM6 final assembly was a combination of DBG2OLC plus sparse, DBG2OLC plus platanus and WTDBG2. Telomeric repeats were searched for using an in-house python script (the script can be found at:

https://github.com/Gabaldonlab/longHam/tree/main/additional_scripts/find_telomeric_repeats.py.).

Short reads from all strains of *N. bracarensis* were then mapped to both reference genomes (CBM3 and CBM6) using PerSVade v1.02.4 [44] to call single nucleotide polymorphisms (SNPs), structural variants and gene coverage which was used to identify duplications and losses.

### Gene prediction and adhesin annotation

Genes were predicted for the long-read assemblies of strains CBM3 and CBM6. First exonerate was used to search for all proteins found in the YGOB [54] database. RATT (downloaded in 2018) from the PAGIT v1 package [55] was then used to transfer the annotation from a previously annotated version of *N. bracarensis* [3]. MAKER v2.31.10 [56] and YGAP web-server (accessed November 2022) [57] were also used to obtain ab-initio predictions. The four gene prediction sources were then joined using EVM (downloaded in 2018) (programs / parameters: partition_EVM_inputs.pl: --segmentSize 100000 --overlapSize 10000; write_EVM_commands.pl with equal weights for all predictions; execute_EVM_commands.pl; recombine_EVM_partial_outputs.pl; convert_EVM_outputs_to_GFF3.pl) [58].

Once the gene prediction was finished we used a pipeline previously designed to predict adhesins (see methods in [24]). The presence of a GPI-anchor was also verified using NetGPI v1.1 [33]. A secretion signal was also searched for using SignalP 6.0 [59]. Based on the set of proteins predicted with this pipeline we searched for motifs of 5, 6 or 7 base pairs that were highly repeated in adhesins, similarly to the VSHITT and SFFIT motifs found in *N. glabratus*. One such motif was found in *N. bracarensis* adhesins (SDGKTHT). To ensure the motif was specifically expanded for adhesins we searched for it in proteins of the OMA database (version June 2019) [34]. Proteins that contained this repeated motif and had not been included by the previous pipeline were added to the collection of adhesins. If adhesins were not a part of the annotation build with EVM they were manually added to the annotation.

To evaluate the relative location of the motifs within the protein, we located the starting position of each repeated motif and then divided this position by the total length of the sequence. A density plot was then plotted to see whether any preference could be observed (see Supplementary Fig. 2). O-linked glycosylation was analysed using the web server of GlycoEP [35], a tool designed to detect glycosylation patterns in eukaryotic sequences. The mode used was Prediction based on Composition profile of patterns (CPP) from the Standard prediction tools. The analysis was performed independently on the set of 31 proteins of CBM3 that contained at least one SDGKTHT, on the remaining set of adhesins, and on 5 different sets of 30 random proteins of CBM3 that excluded adhesins. This results in a list of S / T amino acids with a prediction on whether they are glycosylated or not. Then the number of positions with glycosylated S / T amino acids are divided by the total length of the protein sequence and the average is calculated for each dataset.

### Adhesin families and phylogenetic analysis

A complete set of adhesins was built combining predicted adhesins of the two strains of *N. bracarensis*. Adhesins of *N. glabratus* were added to the collection by selecting a member of each of the families established previously [24]. As different strains of *N. glabratus* contain different adhesins we established a preference list where an adhesin was first selected from the reference strain CBS138 whenever presence, followed by the well annotated BG2 strain. When adhesins were not present in either strain then sequences from the other strains sequenced and annotated in that study were selected. The complete list of selected adhesins for *N. glabratus* can be found in Supplementary Table 4. Sequences were then trimmed so that only their N-terminal region was considered (first 300 aa). This is a methodology that has been adopted to avoid the large repetitive regions adhesins can contain and that hamper the reconstruction of phylogenetic trees. MCL v14-137 [39] was then used to cluster the adhesins into families (Inflation parameter = 1.5). For each family that contained at least 4 sequences a phylogenetic tree was reconstructed as follows. Sequences were aligned using three different programs: MUSCLE v3.8.1551 [60], MAFFT v7.407 [61] and KALIGN v2.04 [62]. Sequences were aligned twice, first in forward and then in reverse [63]. M-coffee v12.00 [64] was then used to obtain a consensus alignment based on the six pre-calculated alignments. This alignment was then filtered using trimAl v1.4.rev15 [65] using a consistency score and a gap threshold (-ct 0.1666666, -gt 0.1 and -cons 30). The final alignment was then used to reconstruct a maximum phylogenetic tree using IQTREE v1.6 [45]. The best model was found using modelFinder as implemented in IQTREE but limiting the search to five different models (DCmut, JTTDCMut, LG, WAG, VT). The number of free rate categories was limited to between 4 and 10. Support was calculated using 1000 rapid bootstraps. ETE v3 [66] was then used to visualize the trees.

## Electronic supplementary material

Below is the link to the electronic supplementary material.

Supplementary Material 1: PDF file including Supplementary Fig. 1, Supplementary Fig. 2 and the legends of the Supplementary figures and Supplementary tables

Supplementary Material 2: Excel formatted file containing the Supplementary tables

## Data Availability

All sequencing data and genome assemblies and annotations can be found in NCBI under bioproject PRJNA1001830.

## Abbreviations

BMT: Beta-mannosyltransferase
DSS: Dextran sulfate sodium
EPA: Epithelial adhesins
SNPs: Single nucleotide polymorphisms
