# Supplementary material for: Chromosome level assemblies of Nakaseomyces (Candida) bracarensis uncover two distinct clades and define its adhesin repertoire
Source: BMC Genomics. 2024 Nov 7;25:1053. doi: 10.1186/s12864-024-10979-8 (PMC11542307; doi:10.1186/s12864-024-10979-8)
Supplement: Supplementary file 1 — Supplementary Material 1: PDF file including Supplementary Fig. 1, Supplementary Fig. 2 and the legends of the Supplementary figures and Supplementary tables [file 12864_2024_10979_MOESM1_ESM.pdf]

Supplementary material for: Chromosome level assemblies of Nakaseomyces (Candida) bracarensis uncover two distinct clades and define its adhesin repertoire

Supplementary Figures

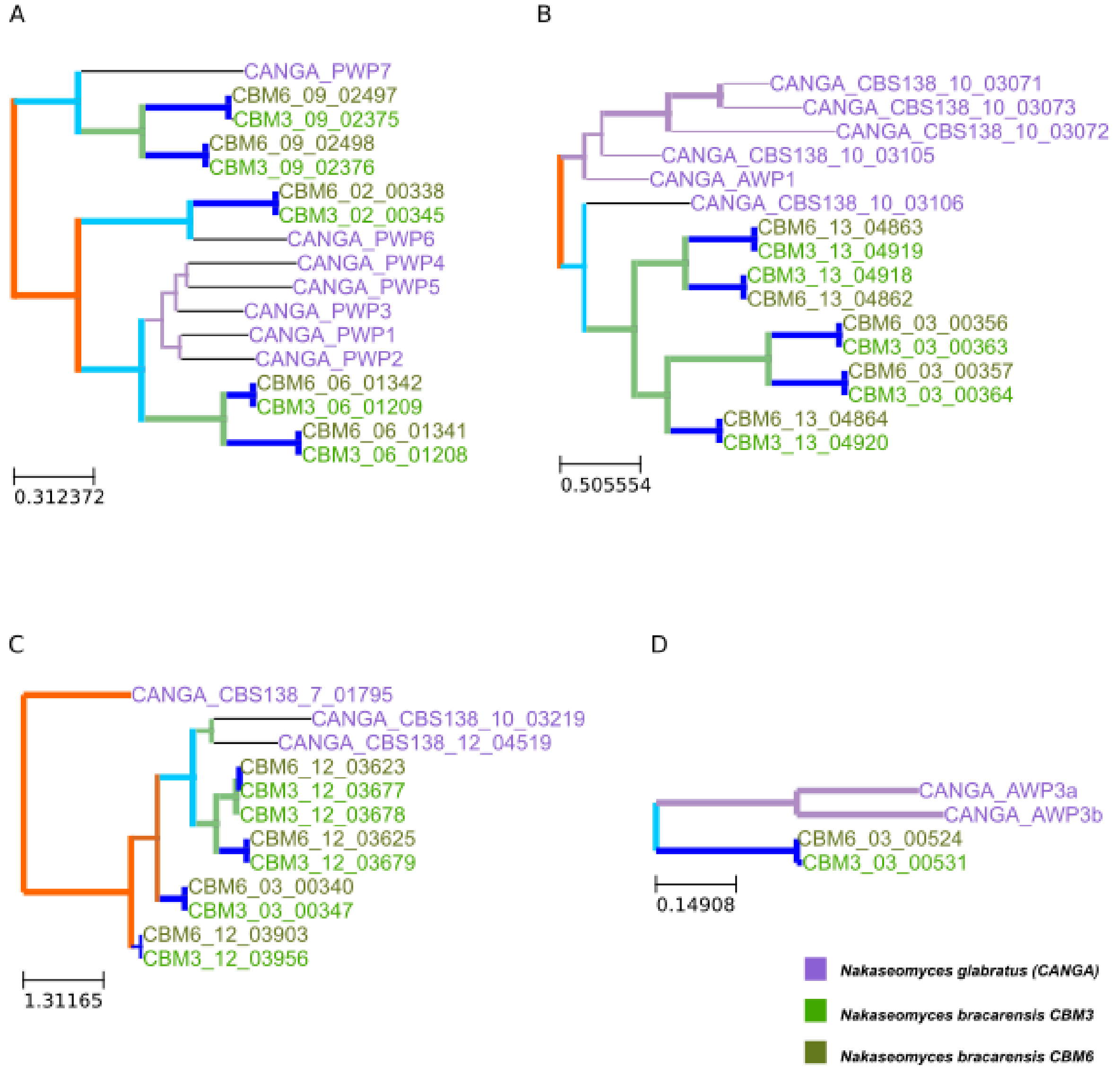

Supplementary Figure 1: Trees depicting the phylogenetic relationship of adhesins found in families 5 (A), 6 (B), 7 (C) and 8 (D). Trees are colored as in Figure 2.

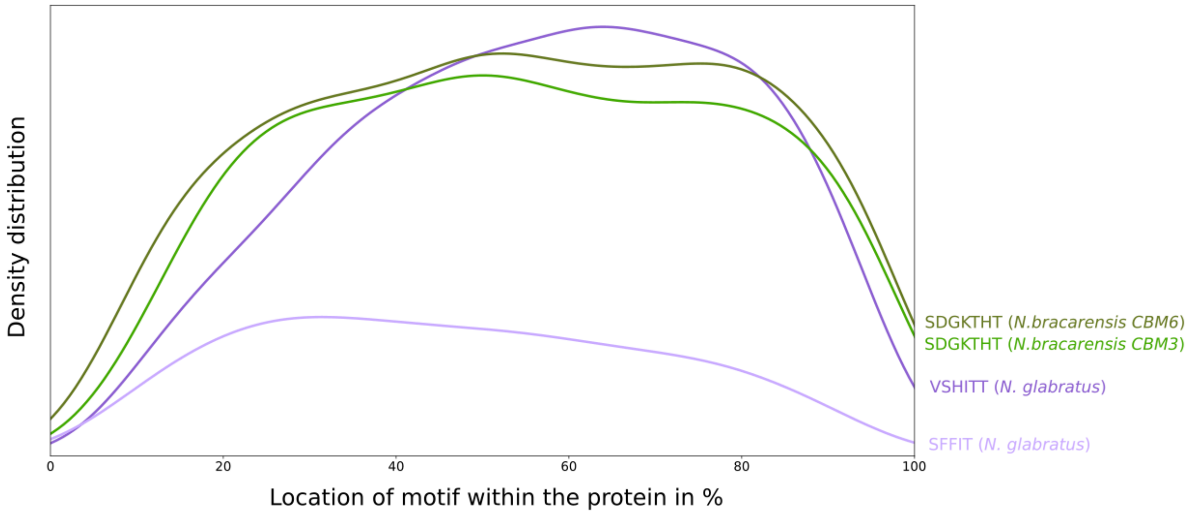

Supplementary figure 2: Density plot representing the occurrence of repetitive adhesin motifs along a protein sequence expressed as a percentage related to the starting point of the motif within the protein sequence. Green colours represent the density plots of the SDGKTHT motif within the two N. bracarensis strains. Purple colours indicate the density plots for the two motifs in N. glabratus (VSHITT and SFFIT).

Supplementary table legends

Supplementary table 1: List of genes in *N. bracarensis* that are missing in at least one of the strains. First column indicates the ID of the gene lost, the second column the genome that has been used as reference to detect the loss, the third one the in which clade or strain the gene has been lost and finally the fourth column lists the putative gene function.

Supplementary table 2: List of predicted adhesins in *N. bracarensis* CBM3. The first column indicates gene ID. The second column indicates whether the current version of the sequence contained a GPI-anchor (noted as GPI-Anchored) or not (noted as a dash). The next three columns indicate the number of times each of the different adhesin motif appears in the sequence (VSHITT, SFFIT and SDGKTHT). Column six indicates whether the adhesin is predicted as secreted and column seven indicates the presence of the PA14 domain which identifies adhesins. Finally the last column shows the family the adhesin belongs to.

Supplementary table 3: List of predicted adhesins in *N. bracarensis* CBM6. Columns are the same as detailed in the legend of Supplementary table 2.

Supplementary table 4: List of adhesins selected from *N. glabratus* for the comparative analysis with adhesins of *N. bracarensis*.
